# Chile (*Capsicum annuum*) plants transformed with the *RB* gene from *Solanum bulbocastanum* are resistant to *Phytophthora capsici*

**DOI:** 10.1371/journal.pone.0223213

**Published:** 2019-10-07

**Authors:** Suman Bagga, Yvonne Lucero, Kimberly Apodaca, Wathsala Rajapakse, Phillip Lujan, Jose Luis Ortega, Champa Sengupta-Gopalan

**Affiliations:** 1 Department of Plant and Environmental Sciences, New Mexico State University, Las Cruces, NM, United States of America; 2 Department of Entomology, Plant Pathology, Weed Science, New Mexico State University, Las Cruces, NM, United States of America; University of Tsukuba, JAPAN

## Abstract

*Phytophthora capsici* is a soil borne pathogen, and is among the most destructive pathogens for *Capsicum annuum* (chile). *P*. *capsici* is known to cause diseases on all parts of the chile plants. Therefore, it requires independent resistance genes to control disease symptoms that are induced by each of the *P*. *capsici* strains. This requirement of multiple resistance genes to confer resistance to *P*. *capsici*, in chile makes breeding for resistance a daunting pursuit. Against this backdrop, a genetic engineering approach would be to introduce a broad host resistance gene into chile in order to protect it from different races of *P*. *capsici*. Notably, a broad host resistance gene *RB* from *Solanum bulbocastanum* has been shown to confer resistance to *P*. *infestans* in both *S*. *tuberosum* and *S*. *lycopersicum*. We agroinfiltrated the *RB* gene into the leaves of susceptible chile plants, demonstrating that the gene is also capable of lending resistance to *P*. *capsici* in chile. We introduced the *RB* gene into chile by developing an *Agrobacterium tumefaciens* mediated transformation system. The integration of the *RB* gene into the genome of the primary transformants and its subsequent transfer to the F1 generation was confirmed by genomic PCR using primers specific for the *RB* gene. A 3:1 ratio for the presence and absence of the *RB* gene was observed in the F1 progeny. In addition to showing resistance to *P*. *capsici* in a leaf inoculation experiment, about 30% of the F1 progeny also exhibited resistance to root inoculation. Our data, when taken together, suggests that the *RB* gene from *S*. *bulbocastanum* confers resistance against *P*. *capsici* in *C*. *annuum*, thereby demonstrating that the *RB* gene has an even broader host range than reported in the literature–both in terms of the host and the pathogen.

## Introduction

*Phytophthora capsici* is a soil borne pathogen that is known to cause root rot, foliar blight and pod rot in different cultivars of *Capsicum annuum* (chile); it is among the most destructive pathogens for chile [1]. Most commercial cultivars of capsicum are either very susceptible or only partially resistant to *P*. *capsici*. According to the gene-for-gene hypothesis [2], disease resistance starts with the specific interaction of the pathogen encoded *avr* gene product with the plant R proteins, which are products of the resistance genes. This is then followed by a signal cascade which leads to a hypersensitive response (HR) and resistance [3]. When both the *R* gene in the plant host and the cognate *avr* are present in the pathogen, the plant pathogen interaction then becomes incompatible. Subsequently, the host exhibits full resistance to the pathogen. Most of the *R* genes encode intracellular proteins carrying leucine-rich repeat (LRR) and nucleotide-binding site (NBS) domains [4]. Generally, it is opined that NBS-LRR R proteins have a modular structure with distinct recognition and signaling domains. While LRR is the recognition site, the NBS region is involved in initiating the signal cascade [5]. Furthermore, the LRR is the most variable region in closely related NBS-LRR proteins and is under selection to diverge [6].

Traditional breeding programs have often been successful at finding resistance genes that are active against single races of a pathogen. In cases where a single race of a pathogen has been found to predominate, this resistance typically lasts for several years until a new race of the pathogen emerges. However, in cases where multiple races of the pathogen are present, as seems to be the case with *Phytophthora*, classical breeding for resistance proves to be a far more challenging endeavor. *P*. *capsici* can cause disease on all parts of the chile plants [7] and requires independent resistance genes to arrest the symptoms of disease induced by each of the *P*. *capsici* strains [8]. Therefore, the requirement of multiple resistance genes for a chile plant to be resistant to *P*. *capsici* makes breeding (for the purpose of disease resistance) in chile a daunting task. Forty five physiological races for *Phytophthora* root-rot and foliar blight disease syndromes have been identified [1] with probably as many different *R* genes [8]. In *C*. *annuum*, only a few resistant cultivars have been identified so far [9], with Criollo de Morelos (CM-334) reporting the highest level of resistance. Some reports have identified at least two resistance genes, while others have highlighted a single dominant gene, or a single dominant gene with modifying genes in CM-334 [9]. Regardless of the number of genes being involved in conferring resistance, it can be surmised that resistance in chile follows polygenetic inheritance. To that end, systematic efforts have been made to identify quantitative trait loci (QTL) associated with *P*. *capsici* resistance. In addition, several molecular markers linked to these QTLs have been reported [9–11]. However, attempts to transfer the QTLs into elite but susceptible chile cultivars have largely been unsuccessful [1]. Moreover, moving resistance genes into susceptible cultivars of chile, using recurrent selection, has been rendered difficult due to the association of linkage drag with low yield, small and undesirable fruit, and less vigorous plants [12].

In addition to all the existing breeding efforts, a genetic engineering approach to confer resistance in chile could potentially be used to combat *P*. *capsici*. There are reports pertaining to the use of *R* genes to confer resistance against specific pathogens [13]. Several *R* genes have been cloned and introduced into different plants and have been shown to confer resistance to a variety of pathogens [13–16]. In this regard, one of the best examples of a broad-spectrum resistance gene is the *RB* gene from *Solanum bulbocastanum* [17] for resistance against *Phytophthora infestans*. The *RB* gene when introduced into cultivated potato (*Solanum tuberosum*) and tomato (*Solanum lycopersicum*), conferred resistance to *P*. *infestans* [18–19]. These transgenic tomato plants showed resistance to *P*. *infestans* isolates derived from potato and those extracted from a diseased tomato plant, with the latter being more aggressive. Given the intrinsic complexity of *Phytophthora* populations, broad-spectrum resistance genes are probably necessary to impart efficacious resistance against *P*. *capsici* in chile. In attempts to find a suitable broad host resistance gene, we tested the efficacy of the *RB* gene from wild potato, to confer resistance in susceptible chile to *P*. *capsici*. Our initial results demonstrate that transgenic chile plants containing the *RB* gene from *S*. *bulbocastanum* do indeed exhibit resistance to *P*. *capsici*.

## Materials and methods

### Agroinfiltration

#### Plant material

Seeds of Mesilla Cayenne (NuMex 6–4) and CM-334 were obtained from the New Mexico State University (NMSU) chile pepper breeding program.

#### Gene construct

*RB* gene construct *pCLD04541*, along with its native promoter, was obtained from Dr. Jiming Jiang [17].

#### Preparation of *Agrobacterium* cultures

We used the protocol developed in our lab to prepare the Agrobacterium culture used for infiltration [20]. Overnight starter culture of *Agrobacterium tumefaciens* that contained either the *RB* gene construct or the empty vector (*Cambia 2300*) was grown on LB media and supplemented with antibiotics (100 mg/L kanamycin) as well as 50 mg of Rifampcin/L) (Kan_100_ and Ref_50_). Starter cultures were then used to inoculate 10 ml LB medium pH 5.7 with 10 mM 2-N-Morpholino ethanesulfonic acid (MES), Kan_100_, Ref_50_, and 20 μM acetosyringone (AS). Thereafter, the cultures were chilled on ice for 10 minutes, pelleted by centrifugation at 4000 rpm (Eppendorf Centrifuge 5810R; Hauppauge, NY) for 5 minutes before being washed twice with LB medium. The pellet was resuspended in induction medium pH 5.7 with 10 mM MES, and 150 μM AS. The cultures were incubated at room temperature for three hours to overnight to a density of ~ 0.5 at OD_600_.

#### Infiltration

The protocol is essentially as per the description of Ortega et al. [20]. NuMex 6–4 seeds were germinated in MetroMix 360 Professional Growing Medium soil (Agawam, MA) under artificial light. Seedlings at the two-leaf stage (roughly four to six weeks after seeds were started) were transferred to four-inch pots for approximately three weeks or more until six mature leaves were present. Uniformly sized, healthy plants were selected for infiltration. Using a 1 ml syringe, 4–5 leaves that were uniform in size were infiltrated on each NuMex 6–4 plant in the 4–6 true leaf stage with *A*. *tumefaciens* containing the *RB* gene and *A*. *tumefaciens* containing the non-recombinant *pCambia 2300* vector (control). Equal numbers of plants were infiltrated with each construct and infiltrated leaves were then marked with loosely-tied thread.

### Chile transformation protocol

NuMex 6–4 seeds were surface sterilized in 90% ethanol for 15 seconds and treated for 10 minutes in 15% (v/v) Chlorox bleach. This was followed by three rinses in sterile distilled water. Seeds were plated on MS media [21] supplemented with 30 g L^-1^ sucrose and 6.5 g L^-1^ agar. They were then maintained at 26 ^o^ C in the dark for 7 to 8 days followed by a regime of 16 hours light and 8 hours dark period for 3–4 days in a plant tissue culture incubator. Cotyledons with attached petioles were cut from 10 to 12 days old chile seedling and placed on precondition media (MP) for two days. Prior to transformation, these plates were placed in an incubator with a regimen of 16 hours in light and 8 hours in the dark at 26 ^o^ C. All the media used are modifications of the MS media and the composition of each media is shown in Table 1. The pre cultured cotyledon explants were transferred to *Agrobacterium* culture and swirled around for 15 minutes. Explants were then briefly dried on sterile sheets of filter paper before being placed on co-culture media MC for two days. The co-cultured explants were washed in MS liquid with ticarcillin disodium 1.0 g L^-1^, blotted dry and transferred to MSC1 media for seven days. Cultures were transferred to MSC2 for further growth and development, with each of them being placed 26 ^o^ C in an incubator for a 16 hours light and 8 hours dark regimen. Cultures were transferred to fresh media every two weeks for three transfers. MSC3 media was used for shoot elongation. Individual shoots were cultured on MSC4 media for root initiation. After being rooted on antibiotic selection, the transgenic shoots were transferred to soil and moved to the green house for seed set.

**Table 1:** Composition of the different media used for chile transformation.

Media	Composition:
MP	MS + B_5_ vitamins + BAP (6-benzylamino purine) 5mg L^-1^ + IAA (Indole-3-acetic acid) 1mg L^-1^ + GA_3_ (gibberellic acid) 1mg L^-1^
MC	MS + B_5_ vitamins + BAP 5 mg L^-1^ + IAA 1 mg L^-1^ + GA_3_ 1mg L^-1^ + AS 200 μM L^-1^
MSC1	MS + B_5_ vitamins + BAP 5 mg L^-1^ + IAA 1mg L^-1^ + GA_3_ 1mg L^-1^ + CW (Coconut water) 5% + AgNO_3_ 5 mg L^-1^ + ticar (ticarcillin disodium) 500 mg L^-1^
MSC2	MS salts with B_5_ vitamins + BAP 5 mg L^-1^ + IAA 1mg L^-1^+ GA_3_ 1mg L^-1^ + CW 5% +AgNO_3_ 5 mg L^-1^ + kan 100 mg L^-1^ + ticar 500 mg L^-1^
MSC3	MS + B_5_ vitamins + zeatin 1 mg L^-1^ + IAA 1 mg L^-1^+ GA_3_ 1.5 mg L^-1^ + AgNO_3_ 3 mg L^-1^ + CW 5% + kan 100 mg L^-1^ + ticar 500 mg L^-1^
MSC4	MS + B_5_ vitamins + IAA 0.1 mg L^-1^+ NAA 0.2 mg L^-1^+ kan 100 mg L^-1^ + ticar 500 mg L^-1^

Chile transformation and regeneration protocols were developed in our lab based on the protocol developed by Liu et al [22]. The major differences between the two protocols are the following: i. The co-culture time of the explant with the agrobacterium culture was two days in our protocol as opposed to four days; ii. We used 100 mg L^-1^ of Kanamycin instead of 50 mg L^-1^; and iii. We included 1 mg L^-1^ zeatin in the shoot elongation media.

### Tobacco transformation

The protocol is in accordance with the descriptions of Seger et al. [26].

### Analysis of disease resistance

#### Inoculum preparation

*Phytophthora* isolates were established on V8 agar following the protocol of Monroy-Barbosa and Bosland [8]. Discs of agar-containing *P*. *capsici* were cut from the V8 media [23] and placed on petri dish containing 25 ml of distilled water. These plates were stored in crisper box and incubated at 28 ^0^ C for twenty-four hours. Dishes were then examined under a microscope. In addition, the plates with considerable sporangia growth were kept at 4 ^0^ C for 30–45 minutes and then placed in the 28 ^0^ C incubator for 15–30 minutes. The contents of the petri dishes were emptied through a funnel lined with cheesecloth into an autoclaved flask, after which a 20 μl sample of inoculum was placed on a hemocytometer and zoospores were counted using the Neubauer chamber counting method. An average of two counts was taken in order to ensure accurate estimation of zoospores. Inoculum was then diluted to appropriate zoospore concentration: ~ 1,000 zoospores/ml for root inoculation and ~500 zoospores/10 μl for leaf assay.

#### Leaf inoculation

Two days after infiltrating with *A*. *tumefaciens* containing the *RB* gene and the control vector, the infiltrated leaves were inoculated with 10 μl of 50 zoospores/μl *P*. *capsici* inoculum. The zoospores were dispensed onto sterilized filter paper for a total of 500 zoospores per leaf which were then placed on the leaf surface [24]. The plants were then kept in a well lighted chamber with humid conditions favorable for *P*. *capsici* growth and stored for 96 hours after being inoculated.

#### Root inoculation

Root inoculation experiments were done essentially as described by Reeves et al [25]. Seeds from non-transformed NuMex 6–4 pepper and *P*. *capsici* resistant CM334 cultivars, along with the F1 seeds (obtained by selfing the primary transformants), were germinated in autoclave-sterilized MetroMix 360 under artificial light in individual one-inch pots. Plants were moved to the green house two days prior to inoculation. Plants were inoculated once the plants reached the four-leaf stage (about 4–6 weeks after seeds were germinated). Five ml of 1,000 zoospores/ml were dispensed into an inch deep hole near the root of each plant, after which the hole was covered with the surrounding soil and the plants were left for monitoring over the next seven days. The soil of the inoculated plants was kept fairly moist and plants were not watered for 48 hours post-inoculation given that *P*. *capsici* is mobilized in water. Pictures were taken seven days after inoculation.

#### Detached leaf assay

Two leaves each from *RB* gene transformed F1 progeny (multiple), in addition to CM334 positive control (+ ve) and nontransformed (NT) NuMex 6–4 pepper as negative control (- ve), were placed in a sterile petri dish. The inoculum with 500 zoospores of *P*. *capsici* on white filter paper discs was placed on leaf samples to indicate the site of initial infection. Observations were made for four days and the pictures were taken 96 hours after inoculation. CM334 was used as a resistant control in all the experiments. Many replicates of this experiment were made using leaves from different plants.

### Molecular analysis

#### Isolation of genomic DNA and RNA

DNA was isolated using the CTAB method [27] and RNA was isolated using the LiCl protocol [28].

#### PCR and RT PCR

Genomic PCR was performed by adopting the standard protocol using specific primer set for the *NPTII* gene and specific primers for the *RB* gene. This was designed using the *RB* gene GenBank accession number (AY336128). (IDT; Coralville, Iowa). The sequence of the primer set used for the *NPTII* gene was 5’- CAGGTTCTCCGGCCGCTTGG-3’ and 5’TCGCCGCCAAGCTCTTCAGC-3’ and 5’-CACCTTTCAGTCCTCCCAATAC-3’ and 5’-CATCCTCTAGCTCCATGTTTCC-3’ for the *RB* gene. For RT PCR, RNA was extracted from the leaves and then quantified using a DU Series 500 spectrophotometer (Beckman; Fullerton, CA). Superscript III First-Strand Synthesis System (Thermo Fisher Scientific; Waltham, MA; Cat: 18080051) used to synthesize cDNA. PCR was conducted and Platinum *Taq* DNA Polymerase (Thermo Fisher Scientific; Waltham, MA; Cat: 10966018). The PCR products were electrophoresed on a 1% (w/v) agarose gel.

## Results

### Examination of the *RB* gene from *S*. *bulbocastanum*, in conferring resistance to *P*. *capsici* in susceptible chile plants using a transient assay

**Fig 1:**
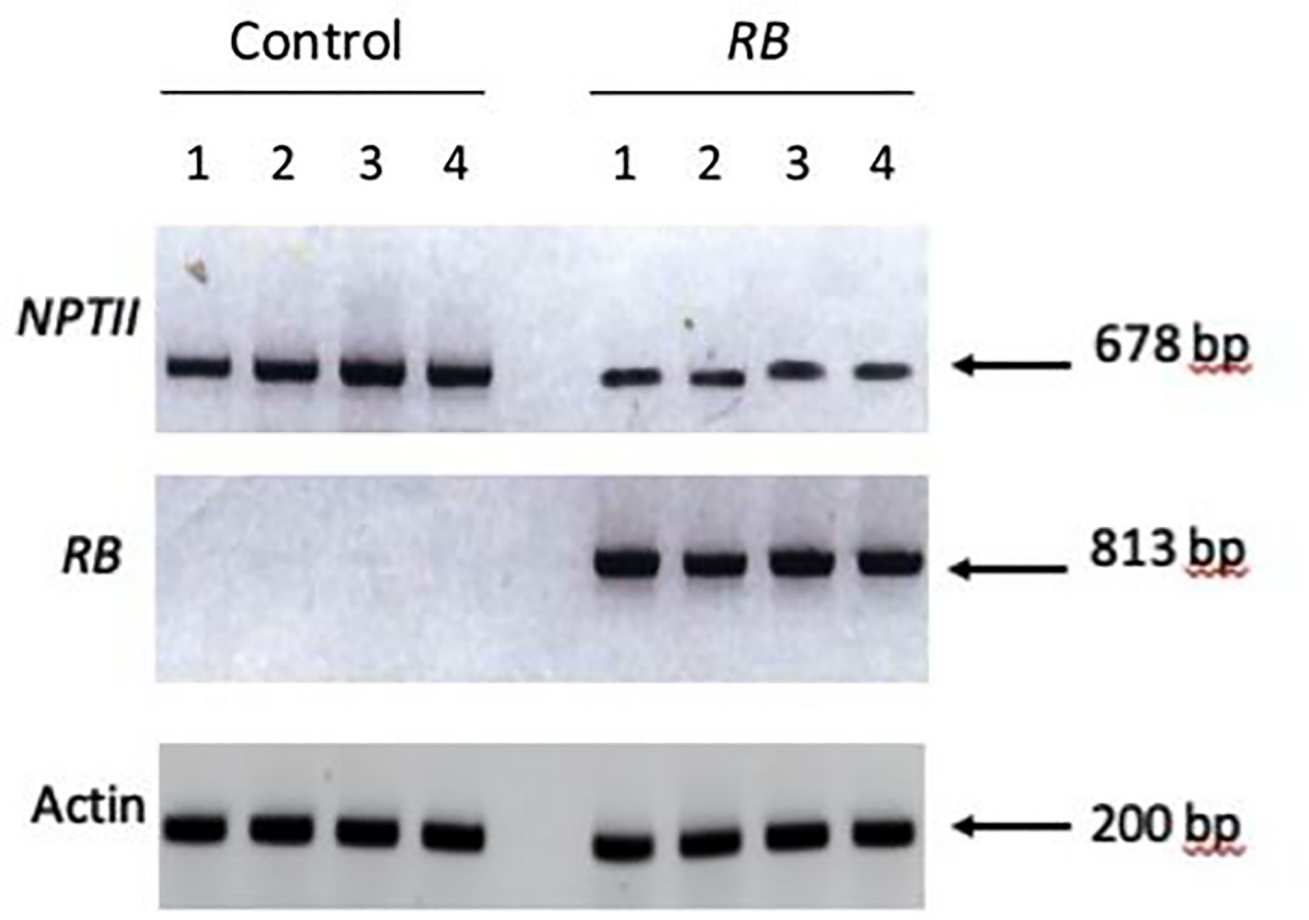
Analysis of expression of the *NPTII* and *RB* genes in the agroinfiltrated chile leaves. Total RNA isolated from the leaves infiltrated with *Agrobacterium* cultures containing either the *RB* gene construct or an empty vector as control was subjected to semi-quantitative RT PCR using specific primers for the *RB* and *NPTII* genes. RT PCR was performed using the *Actin* gene primers as a control for RNA concentration in each sample. The PCR products were then subjected to gel electrophoresis. The size of the amplicons was calculated based on the migration of molecular weight markers.

The *RB* gene from *S*. *bulbocastanum* has been shown to confer broad-spectrum resistance to *P*. *infestans* in cultivated potato and tomato [29]. Since the transformation of chile is very complex and time consuming, we opted to first use agroinfiltration [30–31], to ascertain whether the *RB* gene from wild potato could confer resistance to *P*. *capsici* in chile. Leaves of NuMex 6–4 (*P*. *capsici* susceptible chile) were infiltrated with *Agrobacterium* cultures, while still being attached to the plants. These leaves were infiltrated using *Agrobacterium* containing the *RB* gene construct or *Agrobacterium* with an empty vector as control. In order to check whether the infiltrated *Agrobacterium* cells were transferring their T-DNA into the nucleus and the genes on it were being transcribed, we checked the transcript for the *RB* and *NPTII* genes located on the T-DNA by performing RT PCR on RNA isolated from the agroinfiltrated leaves, using primers specific for the *NPTII* and *RB* genes. RT PCR was performed using the *Actin* gene primers as a control for RNA concentration in each sample. PCR products were subjected to electrophoresis and as illustrated in Fig 1, the amplicon for *Actin* was constant for all the samples, thereby verifying that the quantity of RNA used for RT PCR was the same for all the samples. The leaves infiltrated with *pCambia* 2300 (control) exhibited an amplified product with the *NPTII* primers. Those infiltrated with the gene construct pCLD04541 (*RB*) exhibited a product with *NPTII* and *RB* specific primer sets. Differences were found in the levels of the amplification product for *NPTII* between the plants infiltrated with the *Agrobacterium* containing the empty vector and those with the *RB* gene (Fig 1). This pattern was shown to vary from experiment to experiment, which is probably reflective of the concentration of *Agrobacterium* cells that are infiltrated into the leaves.

**Fig 2:**
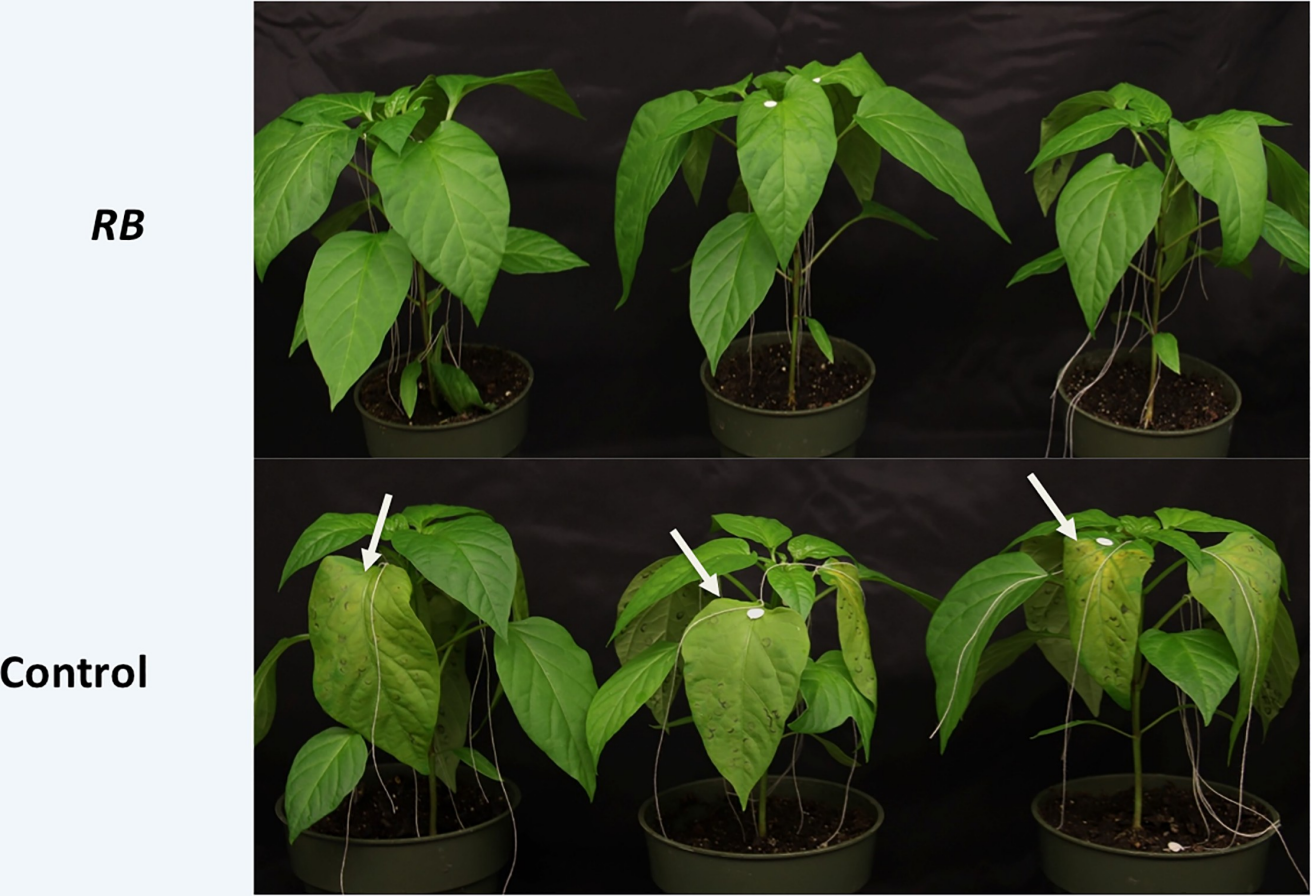
The phenotype of the agroinfiltrated leaves of chile plants that have been inoculated with *P*. *capsici*. Infiltrated leaves of NuMex 6–4 plants with *Agrobacterium* cultures containing either the *RB* gene construct or an empty vector as control were inoculated with *P*. *capsici* zoospores, as described in Materials and Methods section. The leaves were photographed 48 hours post-inoculation. The arrows point to the affected areas on the surface of leaves infiltrated with control Agrobacterium cells.

**Fig 3:**
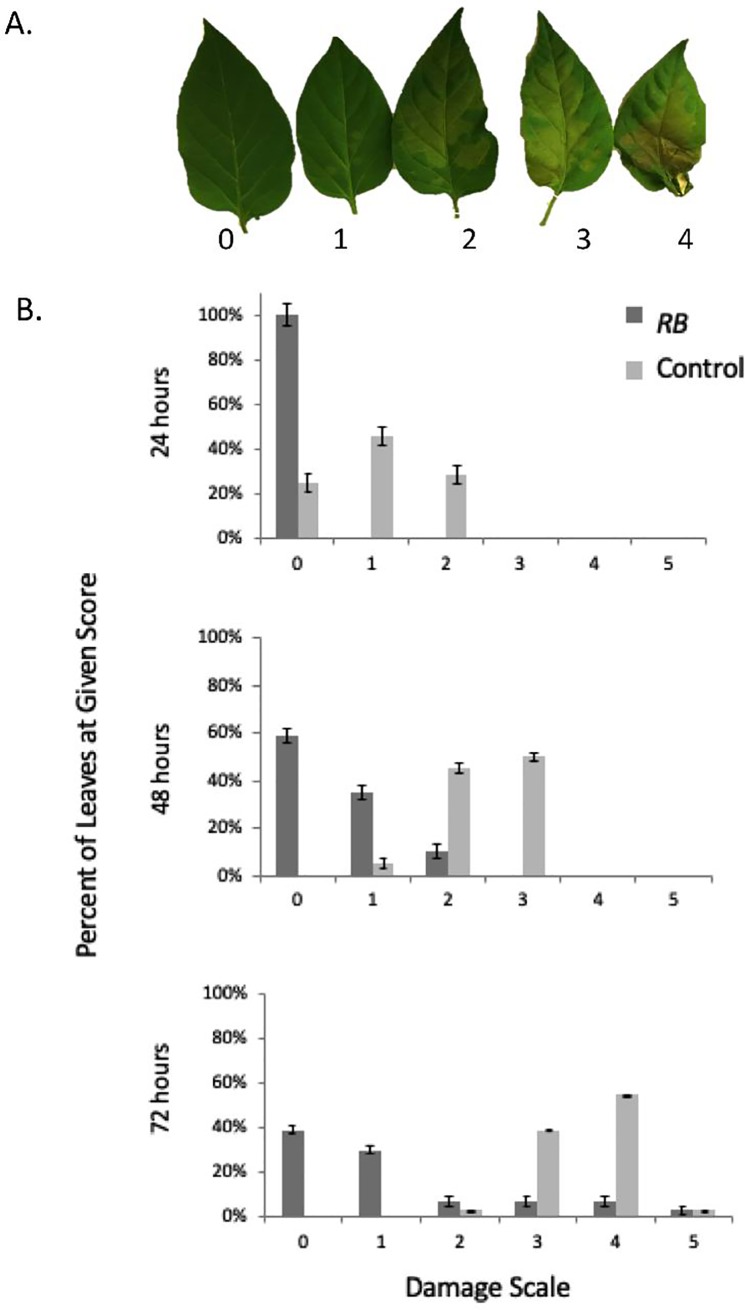
Following the progression of disease in the agroinfiltrated leaves. (A) Fifteen leaves from three independent plants were scored on a damage scale at different times following inoculation where 0 represented a leaf that did not exhibit any disease symptoms and 5 signified a leaf that was completely detached from the plant due to severe disease symptoms. (B) The average damage score for the *RB* infiltrated as well as control leaves is plotted at 24, 48 and 72 hours following inoculation.

Two days following infiltration, the leaves were inoculated with 10 μl inoculum with 500 zoospores that were applied on discs of filter paper 0.5 cm and placed on the top surface of the leaf. The plants were left in a moist chamber and were photographed at 48 hours post inoculation. As evidenced in Fig 2, while all the plants agroinfiltrated with the empty vector did show extensive damage on the leaves, those infiltrated with the *RB* gene did not exhibit disease symptoms. In order to perform an analysis of the progression of the disease symptoms, fifteen leaves from three independent plants were scored on a damage scale at different times following inoculation wherein 0 represented a leaf without any disease symptoms, with a 5 representing a leaf that was completely detached from the plant due to the presence of severe disease symptoms (Fig 3A). As seen in Fig 3B, while the leaves infiltrated with *RB* showed a score of 0 at 24 hours, most of the control plants had already started showing damage up to score 2. By the end of 72 hours, while the control leaves showed damage score of 3 to 4, the leaves infiltrated with *RB* showed damage score of 0 to 1. These results indicate that the *RB* gene does confer some level of resistance to *P*. *capsici* in the susceptible cultivar of chile.

### Developing stable chile transformants (NuMex 6–4) with the *RB* gene construct

**Fig 4:**
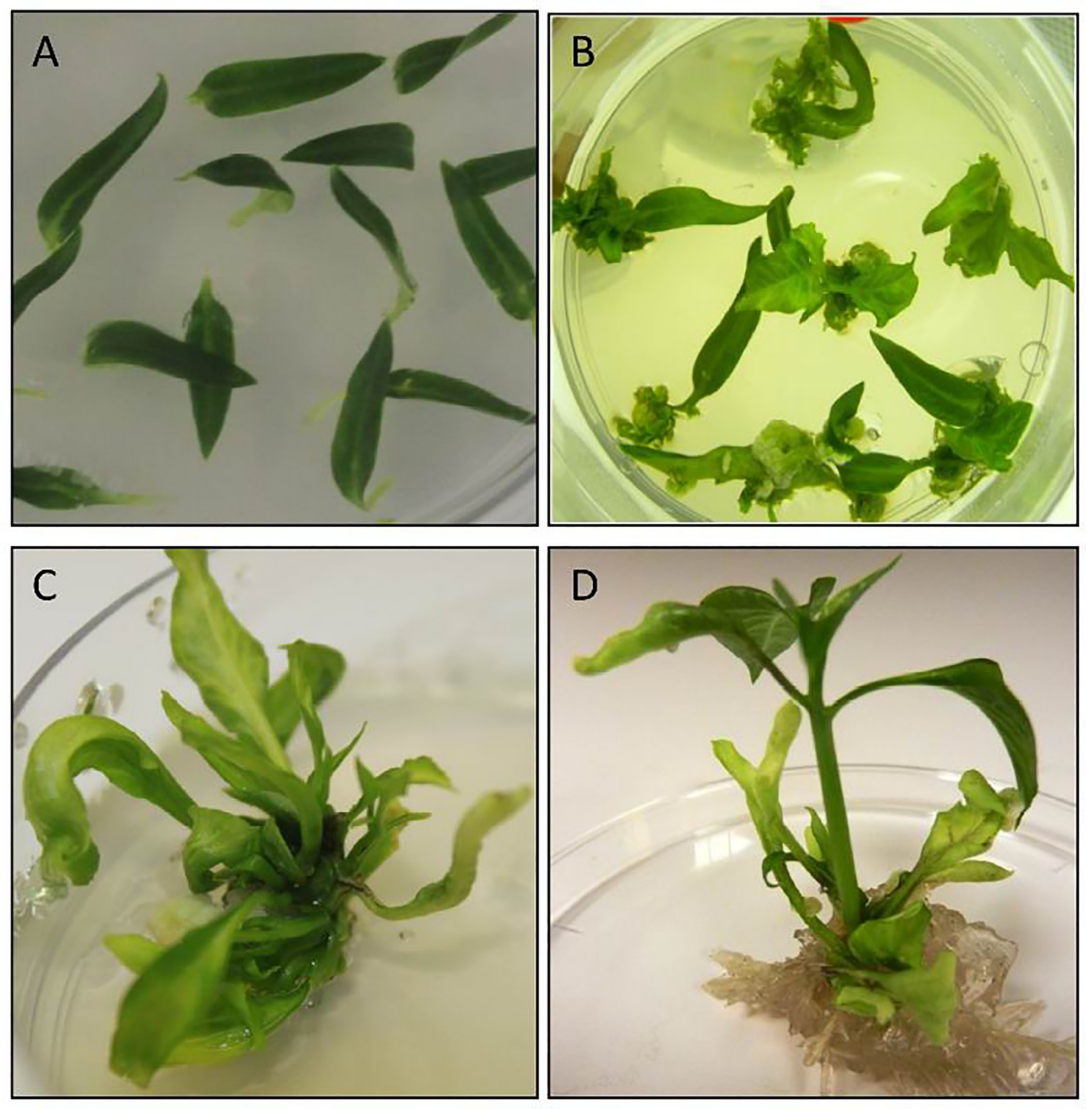
The stages of chile transformation. Chile transformation was performed as described in the section on Materials and Methods. (A) Cotyledons with attached petioles from 10–12 days old seedlings. (B). Cotyledons with calli and leaf clusters on the petioles at 8 weeks. (C) Development of leaves at16 weeks. (D) Shoot elongation and root formation at 24 weeks.

In order to check if the *RB* gene from *S*. *bulbocastum*, would confer stable resistance to whole chile plants, we introduced the *RB* gene into chile (NuMex 6–4) using the transformation and regeneration protocol that was developed in the lab. Chile transformation was undertaken as described in the section in Materials and Methods (Fig 4). The explants produced calli on the media with kanamycin (Fig 4B). Clusters of leaves grew on the callus (Fig 4C). These short clusters of leaves were induced to grow into shoots by growing them in media containing reduced concentration of BAP, IAA and GA_3_ (Table 1) (Fig 4C). The shoots were then moved on to MS media with 0.5 mg L^- 1^ of IAA, where they produced roots (Fig 4D). In addition to using a more stringent selection on kanamycin, we kept the regenerating shoots on 100 mg L^-1^ Kan for root formation. These rooted shoots were moved as individual plants to magenta boxes before being finally transferred to the soil. The average time scale starting from the explant stage to getting established plants in the soil was about 10 to 12 months. Very few shoots rooted on kanamycin. Transformation experiments were conducted multiple times. Based on the frequency of obtaining kanamycin resistant plants, it was inferred that the frequency of transformation was 0.5%. Moreover, many of the putative transformants did not make it to the plant stage. Overall, the efficiency of obtaining whole transformed plants was found to be very poor.

**Fig 5:**
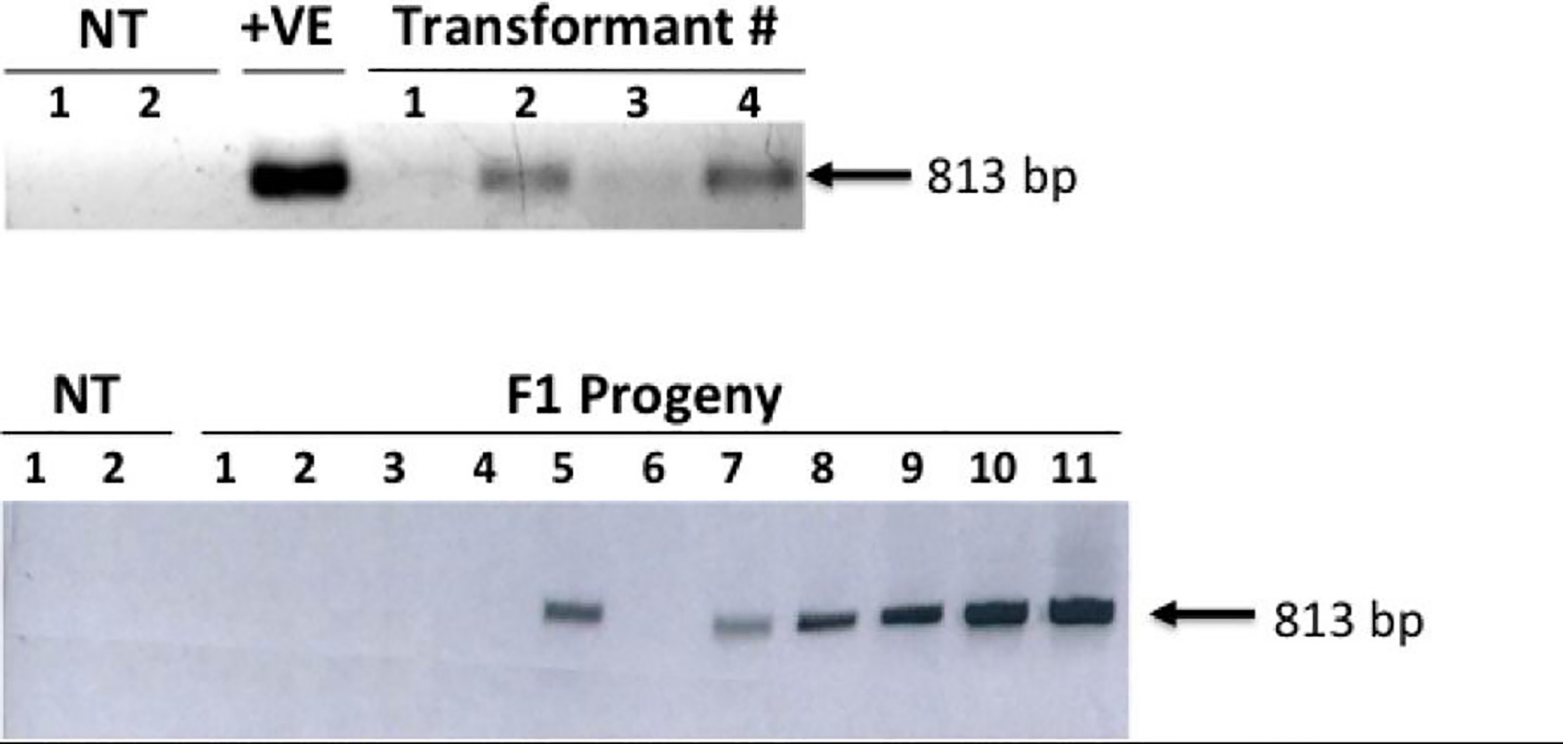
Checking the primary transformants and the F1 progeny for the presence of the *RB* gene. (A) DNA isolated from putative transformants was subjected to genomic PCR using the *RB* gene specific primers. NT denotes non transformed chile plants, and +ve is for DNA isolated from three tobacco plants that were transformed with the *RB* gene. (B) DNA isolated from 10 randomly picked F1 plants produced by selfing the primary transformant #2 and nontransformed chile plants (NT) was subjected to PCR using the *RB*-specific primers. The size of the amplicon is shown.

Four putative transformants were checked by us to ascertain the presence of the transgene. DNA isolated from the leaves of these plants was subjected to PCR using the *RB* gene primer sets. As seen in Fig 5A, only two of the tested plants showed an amplicon of the expected size. These independent transformants were subsequently used for all further studies.

One of the hallmarks of stable transformation is that the transgene is not only properly integrated into the genome of the transformants, but also gets passed on to the successive generations. Seeds collected from the primary transformants following selfing were plated on MS media. Following germination, the seedlings were moved to the soil for further development. DNA was isolated from random plants and subjected to PCR using the *RB*-specific primers. As seen in Fig 5B, three of the 10 randomly picked plants obtained by selfing one of the F1 parents (#2), did not show any amplification. Of the remaining seven plants, two showed higher level of amplification as compared to the rest, probably representing the homozygous individuals with two copies (alleles) of the transgene. However, it is difficult to make such a statement without performing qPCR. Nevertheless, it would appear that the inheritance for the presence/absence of the transgene did follow a Mendelian pattern of 3:1.

### Testing the resistance response in the F1 plants towards *P*. *capsici*

**Fig 6:**
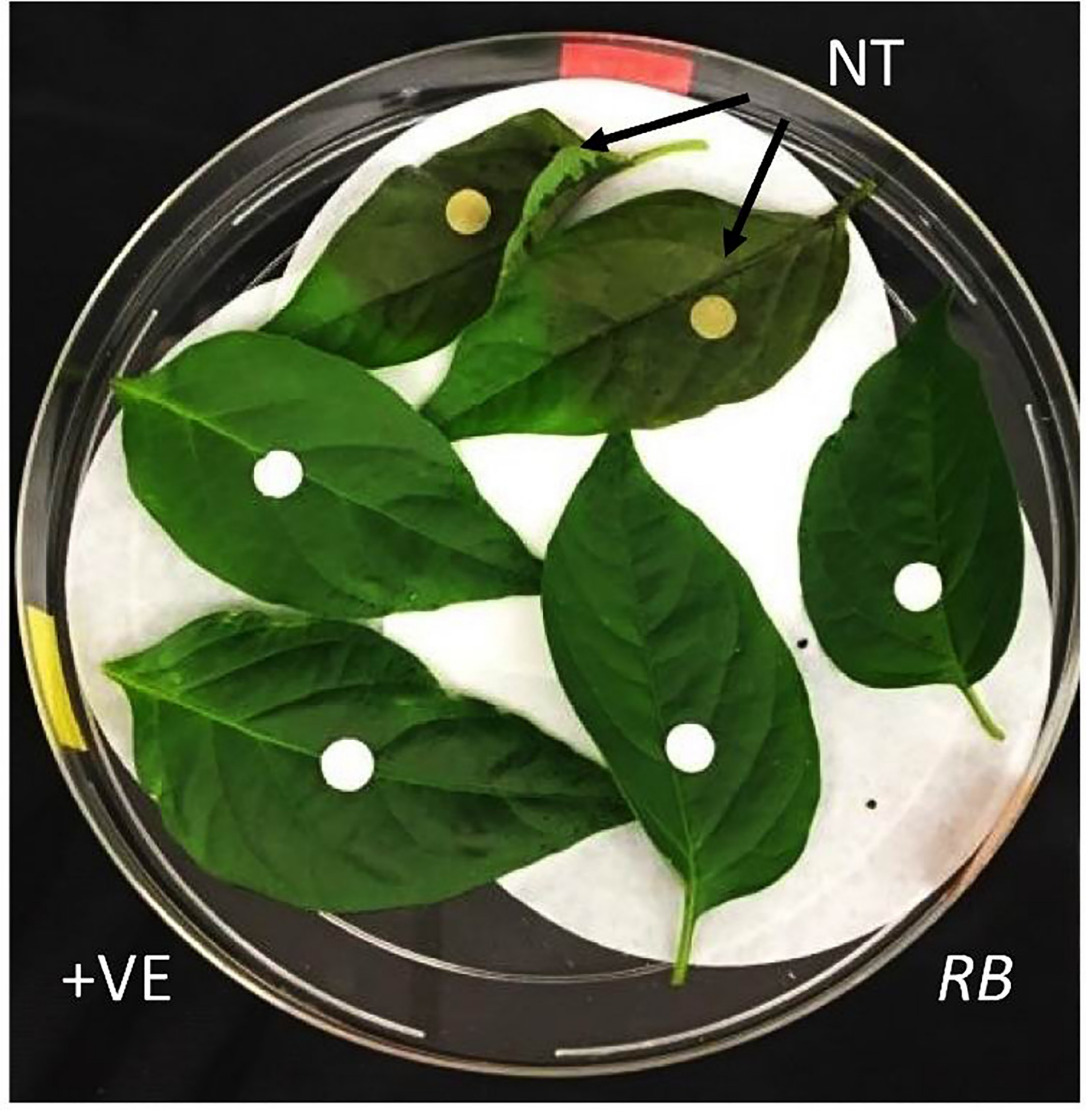
Checking for disease symptoms in the leaves of *RB* transformed plants following leaf inoculation with *P*. *capsici*. Kanamycin resistant F1 plants, along with control plants (NuMex 6–4) and *P*. *capsici* resistant (CM334) cultivar were grown for ~ 1 month in soil. Healthy leaves of the same size from multiple plants (representing the three classes) were plucked and then placed on moist filter paper inside petri dishes. Thereafter, the leaves were inoculated with *P*. *capsici* zoospores as used in the transient assay. A representative picture of two leaves each from a control plant, an F1 plant, and a *P*. *capsici* resistant plant, 72 hours post-inoculation, is shown here. The arrows point in the affected regions on the leaves of NT plants. NT (Control); *RB* (F1 plants); +VE (Resistant).

The seeds of the F1 plants obtained from selfing the primary transformants were germinated on MS media containing kanamycin and tested positive for the presence of the transgene. The seedlings that germinated on kanamycin were moved to soil along with control plants (NuMex 6–4) and *P*. *capsici* resistant (CM334) cultivar. Leaves of the same size from multiple plants, representing the three classes, were plucked and then placed on moist filter paper inside petri dishes. Subsequently, the leaves were inoculated with *P*. *capsici* zoospores, as used in the transient assay. A representative picture of two leaves each from a control plant, an F1 plant with the transgene, and a *P*. *capsici* resistant plant,72 hours post-inoculation, is shown in Fig 6. While the leaves from the control plants did exhibit severe damage (score of 4), the leaves from the transformed plants and the resistant plants did not show any visible damage.

**Fig 7:**
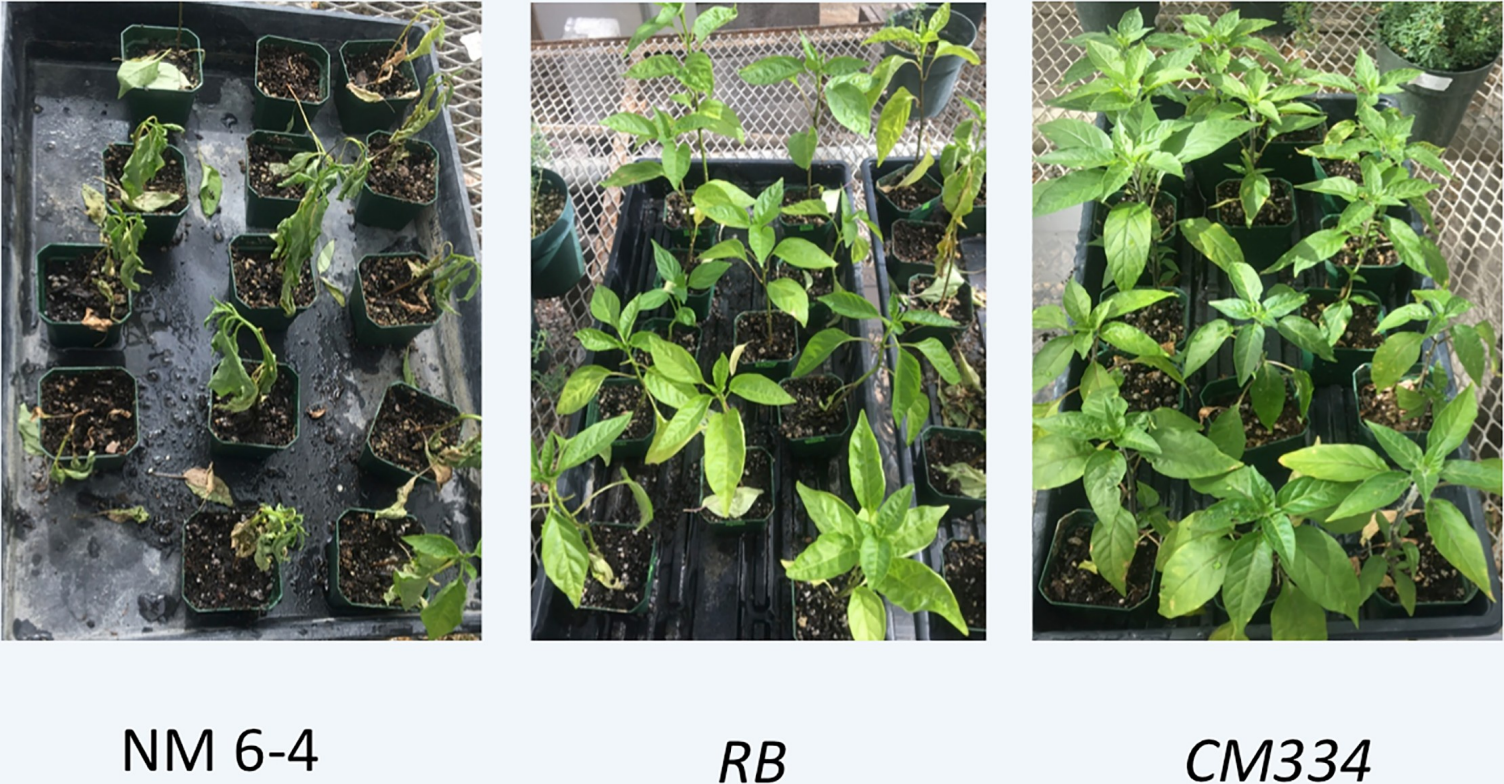
Checking disease development in the F1 progeny of the primary *RB* transformants, following root inoculation with *P*. *capsici*. Seeds of control plants, resistant (CM334) plants, and F1 plants (*RB*) were germinated in small pots (2” X 2”) that were placed on big trays (20” X 10”) before being subjected to root inoculation. Two weeks following inoculation, the healthy versus dead plants were scored and a representative set of trays was photographed. The dead plants from the tray with the *RB* plants were removed in order to highlight the phenotype of the surviving plants.

In order to determine whether the *RB* gene in chile provides resistance against root rot, we used root inoculation on whole plants and monitored the survival rates of the plants. Seeds of control plants, resistant (CM334) plants, and F1 plants were germinated in small pots and allowed to grow for four to six weeks before being subjected to inoculation as, described in Materials and Methods section. The F1 plants were not selected on kanamycin. At 14 days post inoculation, these plants were monitored for disease symptoms and survival/death (Fig 7). It was found that 100% of the control plants were dead, while 100% of the resistant plants did not exhibit any disease symptoms. At this point in time, 25% of the F1 plants were dead, 45% exhibited milder symptoms, while 30% were disease free. The disease free F1 plants could be homozygous for the *RB* gene.

## Discussion

Our results show that the *RB* gene from *S*. *bulbocastanum* not only confers resistance to *P*. *infestans* in *Solanum* species [29], but also in *Capsicum annuum* against *P*. *capsici*. This suggests that the *RB* gene has an even broader host range and is more expansive in terms of not only *Phytophthora* races, but also species. Several resistance genes from species in Solanaceae such as the *Pto* gene of tomato [32] and the *Bs2* gene of pepper [33] were successfully transferred as transgenes into other Solanaceous species and shown to confer resistance to the corresponding pathogen.

Transient gene expression using agroinfiltration has been used fairly extensively, given that it is simple and rapid. Moreover, it can be used to address several different functions [34]. In *Agrobacterium* mediated transformation of plant cells, the genes on the T-DNA are transported into the nucleus where it gets integrated into the genome. The transformed cells are not grown into whole plants in the transient assay; instead, the cells can be analyzed for expression directly post-agroinfiltration. The genes on the T-DNA are expressed at an early stage prior to and independent of the integration event [35]. Since transformation of whole plants is usually a long and complex process, especially for chile, we opted to use the transient assay as the first approach to determine whether the *RB* gene from wild potato would confer resistance against *P*. *capsici*. Agroinfiltration was used to study the interaction between the *avr* gene products of the pathogen with that of the host encoded *R* gene product [34, 36–37]. In order to ensure that the genes on the T-DNA were expressed following infiltration, the infiltrated leaves were harvested two days post infiltration and tested for the transcripts for *NPTII* and the *RB* genes. The *NPTII* transcripts was seen in both sets of leaves, although the levels were found to be higher in the leaves infiltrated with the *Agrobacterium* cells containing the empty vector; this could be ascribed to the differences in the number of *Agrobacterium* cells infiltrated stemming from the difference in the concentration of the cells in the infiltrated media. Moreover, some differences were noticed among the different leaves infiltrated with the same *Agrobacterium* cell suspension, which could be attributed to the size of the leaves or their position on the plant, as was reported earlier [38]. Nevertheless, our data on agroinfiltration suggests that the *RB* gene does indeed confer resistance to *P*. *capsica* in susceptible chile.

Establishing that the *RB* gene from *S*. *bulbocastanum* when introduced into the leaves of chile, conferred resistance to *P*. *capsici*, was a good start. While this in itself is an important finding, our endeavor to produce chile plants that are resistant to *P*. *capsici* has several practical implications. This in turn necessitated the production of whole plants, with the *RB* gene stably integrated in its genome. While many Solanaceous plants were fairly simple to transform, chile is rather recalcitrant to both transformation and regeneration [1]. At this stage, it is beyond comprehension to offer a plausible explanation as to why it is difficult to produce transformed chile plants. The agroinfiltration experiment has already demonstrated that chile cells can be transformed using *A*. *tumefaciens*. Regeneration of plants from chile cells also takes place on a fairly routine basis. Producing transformed plants entails coordinating the processes of transformation and regeneration, such that the transformed cells grow into whole plants. The low efficiency in producing whole transformed plants could be ascribed to fact that this coordination has not yet ben standardized. Another possibility could be that the chile genome is in a state of flux due to the high incidence of transposable elements. In fact it has been shown that 81% of the *Capsicum* genome consists of transposons [39]. Although we are attributing poor transformation efficiency to more frequent transposition events, we currently lack evidence to back this claim and are unaware of any way to test it. After several attempts to produce transgenic chile plants, we could produce only two stable transformants that not only exhibited the presence of the transgene in the primary transformants, but also in the F1 progeny. Approximately 75% of the tested F1 plants did reveal the presence of the transgene, suggesting Mendelian inheritance.

While the leaves from control plants displayed extensive necrosis in the leaf inoculation experiment, the F1 plants (with the transgene) showed no signs of necrosis till at least 96 hours post inoculation, similar to the leaves from the resistant cultivar, CM334. In the root inoculation experiment, seeds obtained by selfing the primary transformants, as well as those of NuMex 6–4 and CM334, were germinated. The plants were then subjected to root inoculation. While the control plants died within 3–5 days post inoculation, ~30% of the F1 plants managed to survive disease free past seven days when the experiment was terminated. These results suggest that the *RB* gene protects the plant against both leaf blight ad root rot. This point assumes significance, given that *S*. *tuberosum* (potato) plants containing the *RB* gene, did not show resistance in the tuber despite exhibiting foliar resistance [40].

Though preliminary, our data suggests that the *RB* gene could confer some level of resistance to *P*. *capsici*. Considering the fact that we used only one race out of a multitude of races present in the *P*. *capsici* population, we are currently unsure whether the *RB* gene will be effective in interacting with the other races and provide full protection against the pathogen.

The most direct and non-controversial approach to confer resistance to pathogens in plants is through traditional breeding, which has been improved with the use of molecular markers. Despite all the molecular markers developed for chile so far [9, 11, 41], breeding for *P*. *capsici* resistance has remained difficult. Our approach of introducing a broad host resistance gene from wild potato appears to have conferred some level of resistance to *P*. *capsici*. However, it is possible that the resistance afforded by the *RB* gene could be destroyed in the course of time due to the changes occurring in the *avr* genes. It would be ideal if more broad host resistance genes for *P*. *capsici* could be identified, and multiple resistance genes could be introduced in concert in the susceptible chile plants (gene stacking) [42–43]. The introduction of random mutations in the LRR domain of a resistance gene for *P*. *infestans* (*R3a*) allowed for modulating the specificity of the protein to diverse *avr* products [44]. Coupled with the knowledge of pathogen effector diversity, this strategy can be used to develop synthetic R genes with a broad host range. Alternatively, if it was possible to identify the unique amino acid residues in the LRR region of the *RB* gene that recognizes multiple *avr* gene products, the CRISPR/Cas9 technology could potentially be used to target and edit the sites in the LRR region of the endogenous *R* genes in susceptible chile [45–46].

For all the genetic engineering approaches including gene editing that can be utilized to make *P*. *capsici* resistant chile, requires an efficient transformation system. While the transformation system for chile is very inefficient, we were successful in obtaining a few independent transformants using NuMex 6–4. After having established that the *RB* gene has been integrated in the genome, this gene can be moved to different chile cultivars by breeding.
